# Transient Neonatal Diabetes due to a Mutation in KCNJ11 in a Child with Klinefelter Syndrome

**DOI:** 10.4274/jcrpe.4807

**Published:** 2018-02-26

**Authors:** Amanda R. Dahl, Radhika Dhamija, Alaa Al Nofal, Siobhan T. Pittock, W. Frederick Schwenk, Seema Kumar

**Affiliations:** 1 Mayo Clinic, Department of Pediatric and Adolescent Medicine, Rochester, Minnesota, USA; 2 Mayo Clinic, Department of Clinical Genomics, Phoenix, Arizona, USA; 3 University of South Dakota, Sanford Children Specialty Clinic, Division of Pediatric Endocrinology, Sioux Falls, South Dakota, USA; 4 Mayo Clinic, Department of Pediatric and Adolescent Medicine, Division of Pediatric Endocrinology, Rochester, Minnesota, USA

**Keywords:** Neonatal diabetes, Klinefelter syndrome, KCNJ11

## Abstract

Klinefelter syndrome is the most frequent chromosomal aneuploidy in males occurring in about 1 in 660 males. Epidemiological studies have demonstrated increased risk of type 1 diabetes and type 2 diabetes in adults with Klinefelter syndrome. There is only one previous report of neonatal diabetes in a patient with Klinefelter syndrome. We report transient neonatal diabetes due to a pathogenic heterozygous variant in KCNJ11 in a male infant with Klinefelter syndrome. A 78-day old male infant was noted to have sustained hyperglycemia with serum glucose ranging between 148 mg/dL (8.2 mmol/L) and 381 mg/dL (21.2 mmol/L) three days after undergoing a complete repair of an atrioventricular defect. Hemoglobin A1c was 6.6%. The patient was born at term with a birth weight of 2.16 kg following a pregnancy complicated by gestational diabetes that was controlled with diet. The patient was initially started on a continuous intravenous insulin drip and subsequently placed on subcutaneous insulin (glargine, human isophane and regular insulin). Insulin was gradually decreased and eventually discontinued at seven months of age. Chromosomal microarray at 11 weeks of age showed XXY and a panel-based, molecular test for neonatal diabetes revealed a pathogenic heterozygous variant c.685G>A (p.Glu229Lys) in KCNJ11. The patient is now 34 months old and continues to have normal fasting and post-prandial glucose and HbA1C levels. The patient will need prospective follow up for assessment of his glycemic status. To our knowledge this is the second reported case of neonatal diabetes in an infant with Klinefelter syndrome and the first due to a mutation in the KCNJ11 in a patient with Klinefelter syndrome.

## What is already known on this topic?

Klinefelter syndrome is the most frequent chromosomal aneuploidy in males occurring in about 1 in 660 males. Epidemiological studies suggest increased risk of type 1 diabetes and type 2 diabetes in adults with Klinefelter syndrome. There is only one previous report of neonatal diabetes in a patient with Klinefelter syndrome.

## 

### What this study adds?

To our knowledge, this is the second reported case of neonatal diabetes in an infant with Klinefelter syndrome. This case is the first due to a mutation in the KCNJ11 as the previously reported case of transient neonatal diabetes and Klinefelter syndrome had uniparental heterodisomy of chromosome 6.

## Introduction

Klinefelter syndrome (KS) is characterized by a 47XXY genotype and is the most frequent chromosomal aneuploidy in males occuring in about 1 in 660 males (1). Epidemiological studies have demonstrated increased risk of type 1 diabetes and other autoimmune diseases in adults with KS (2,3,4). Recent studies have also shown an increased risk of type 2 diabetes with accumulation of body fat and a concomitant decrease in insulin sensitivity in KS (5,6). There is only one previous case report of neonatal diabetes in a child with KS (7).

Neonatal diabetes is a monogenic form of diabetes, with an estimated incidence of 1 in 100,000 to 1 in 260,000 live births (8,9,10,11). It is defined as persistent hyperglycemia occurring in the first six months of life that lasts more than two weeks and requires insulin for management (9,12,13). Transient neonatal diabetes mellitus (TNDM) represents 50-60% of cases of neonatal diabetes, with the other 40-50% being permanent neonatal diabetes mellitus (14). The majority of cases of TNDM have paternal uniparental disomy of chromosome 6 or an unbalanced duplication of paternal chromosome 6 (15,16,17). Less frequent genetic abnormalities noted in patients with TNDM include activating mutations in the ATP-sensitive K^+ ^channel encoding genes (*KCNJ11 *and* ABCC8*)(12). We report the first case of TNDM due to a mutation in the *KCNJ11 *gene in an infant with KS.

## Case Report

A male infant was born at 39+3 weeks gestation via non-spontaneous vaginal delivery with a birth weight of 2.16 kg and length of 48 cm. Pregnancy was complicated by gestational diabetes that had been controlled with diet and labor was induced for intrauterine growth restriction. There were no complications in the neonatal period. External genitalia were normal. The infant was noted to have bilateral inguinal hernia at one month of age and underwent bilateral inguinal hernia repair at age 39 days. In the postoperative period, he developed tachypnea and respiratory distress. Physical examination was significant for a loud S1/S2 with no splitting of S2 and a harsh 2-3/6 systolic murmur, loudest at the left sternal border. Echocardiogram revealed a type A complete atrioventricular canal defect with moderate regurgitation of left and right sides of the common atrioventricular valve, multiple ventricular septal defects and a small secundum atrial septal defect. At age 42 days, a random blood glucose level was found to be 175 mg/dL and repeat blood glucose estimation on day 43 was 190 mg/dL. The mild hyperglycemia was attributed to stress. Furosemide and digoxin were started to improve acute heart failure. Subsequently, the infant exhibited poor weight gain and poor feeding. He underwent complete atrioventicular canal repair and secundum atrial septal defect closure at age 74 days. Postoperatively, on day 74, blood glucose values were noted to be between 148 mg/dL and 381 mg/dL. The patient was extubated by post-operative day three and weaned off vasopressors and inotropes by post-operative day four. Hyperglycemia persisted and hemoglobin A1C was noted to be 6.6%. C peptide was 2.7 ng/mL (reference range 1.1-4.4 ng/mL). Family history was significant for diabetes in father since his 20s who was successfully treated with metformin. Additionally, a maternal uncle had been diagnosed with diabetes at age 18 years and he was now on insulin but had been on oral hypoglycemic agents previously.

Intravenous insulin infusion was started on day 78 at an initial dose of 0.03 units/kg/hour and was substituted with subcutaneous insulin injections (insulin glargine and regular insulin) at age 91 days. Though insulin glargine is not approved for use in infants, the release pattern of insulin glargine with no “peaks” makes it attractive during early infancy when infants are feeding frequently. There have been several reports regarding use of insulin glargine in infants with neonatal diabetes (18,19,20). Maximum hemoglobin A1c (HbA1C) was 7.2% at age five months. Glargine was subsequently replaced with intermediate acting isophane insulin due to insurance coverage reasons. Due to improvement in glucose values, insulin doses were gradually decreased and insulin was eventually discontinued at seven months of age. Oral sulfonylurea would be the preferred treatment in our patient given the KCNJ11 mutation. We planned to switch the patient from insulin to oral sulfonylurea after the results of the genetic analyses became available but elected not to do so when the insulin requirements began to decrease. We were able to discontinue insulin successfully with normal serum glucose values after discontinuation of insulin.

Fasting and post-prandial blood glucose values as well as HbA1C have subsequently been monitored and have been in the normal range. HbA1C was 6% at eight months of age, 6.2% at 11 months, 5.7% at 14 months, 5.4% at 19 months and 5.5% at 25 months of age. At the most recent follow up at 31 months of age, fasting glucose was 93 mg/dL and HbA1C was 5.4%. The patient has been gaining weight and growing normally and continues to make slow developmental progress. He started walking at 19 months of age and began receiving speech therapy for speech delay.

### Genetic Analysis

Chromosomal microarray was performed at 11 weeks of age due to the complete atrioventricular canal defect (Cytogenetics Laboratories, Mayo Clinic, Rochester, MN). Chromosomal microarray revealed gain of the entire X chromosome. Limited chromosome study confirmed a diagnosis of KS/47, XXY. As external genitalia were unremarkable, gonadotropins and testosterone levels were not measured. The platform (Affymetrix CytoScan HD platform) used for microarray laboratory was single-nucleotide polymorphism based and did not detect stretches of homozygosity that would lead to possibility of uniparental disomy of chromosome 6. The laboratory was specifically asked to look for homozygosity at 6q24. Simultaneously, sequence analysis of 27 genes associated with neonatal diabetes was performed at the Genetic Services Laboratories of University of Chicago (neonatal diabetes/Maturity Onset Diabetes of the Young sequencing panel). This panel based testing revealed a pathogenic heterozygous variant c.685G>A (p.Glu229Lys) in KCNJ11. There was also a heterozygous variant of unknown significance, c.713G>A (p.Arg238Gln) in *BLK*. Other genes included in the panel were *ABCC8, AKT2, CEL, CISD2, CP, EIF2AK3, FOXP3, GATA6, GCK, GLIS3, GLUD1, HADH, KCNJ11, KLF11, INSR, INS, IER3IP1, NEUROD1, NEUROG3, PAX4, PDX1, PTF1A, RFX6, SLC2A2, WFS1, *and* ZFP57*.

Genetic testing for parents was recommended. The rationale for testing was discussed with the parents, as they may also have the KCNJ11 mutation detected in the patient and how this would influence their own medical care, but they have declined testing.

## Discussion

We report the second case of TNDM in an infant with KS. Genetic testing in this patient revealed a pathogenic heterozygous variant in KCNJ11. This is the first reported case of neonatal diabetes due to a mutation in the KCNJ11 in a patient with KS.

The *KCNJ11 *encodes Kir6.2 subunit of the ATP-sensitive potassium channel in several tissues including pancreatic b cells, brain, heart and skeletal muscles (9,14). Mutations in KCNJ11 lead to a permanent opening of the potassium channel in the pancreatic b cells, thus preventing any activation of voltage-dependent calcium channel and glucose-induced insulin secretion leading to diabetes (21,22,23). The results of the genetic testing in this patient are noteworthy since heterozygous activating mutations in KCNJ11 are seen in only a small number of patients with TNDM (12) and instead comprise the most common cause of permanent neonatal diabetes (24,25,26). However, the pathogenic heterozygous variant c.685G>A (p.Glu229Lys) in *KCNJ11* found in this patient has been previously associated with TNDM (27).

Mutations in KCNJ11 are *de novo* in 80% of cases and inherited in an autosomal dominant pattern in the remaining cases (28). Family history in our patient was significant as his father had been diagnosed with diabetes since his 20s and he was doing well on metformin. Additionally, there was a history of gestational diabetes in the patient’s mother and diabetes requiring insulin therapy in a maternal uncle. Unfortunately, inheritance of the KCNJ11 variant in our child could not be determined as parents have declined testing. We continue to revisit with the parents with our recommendations on parental genetic analysis for better understanding of the significance of the KCNJ11 mutations in our patient. In contrast to our patient, the previously reported case of TNDM and KS had uniparental heterodisomy of chromosome 6 (7), the most common abnormality found in children with TNDM (7,15,16,17). Our patient did not have evidence for uniparental disomy on chromosome 6.

There are several similarities between our case and the previous report (7). Both infants had low birth weight as is expected in children with neonatal diabetes (8). The age at discontinuation of insulin was also similar, with insulin being discontinued at six months of age in the previously reported case and at seven months in our patient (7). Follow up data on both patients is limited and is available only until age two and a half years. These data suggest that there has been no recurrence of hyperglycemia. 40% of patients with TNDM have recurrence of hyperglycemia later in life (8,17,29). Additionally, the hypogonadism associated with KS may lead to changes in body composition and a risk of developing metabolic syndrome and type 2 diabetes (2,6). Therefore, prospective follow up for assessment of glycemic status is warranted in our patient. If hyperglycemia were to recur, this patient would be a candidate for oral sulfonylurea therapy as oral sulfonylureas have been shown to result in improved glycemic control in patients with diabetes due to KCNJ11 mutations (9,23,30).

Another remarkable feature in our patient was the presence of an atrioventricular canal defect, ventricular septal defects and an atrial septal defect. Congenital cardiovascular anomalies are quite uncommon in children with KS. Those that have been reported include Tetralogy of Fallot and transposition of the great arteries (31,32). Parental genetic analyses will lead to better understanding of the significance of the KCNJ11 mutation.
